# On-Lattice Simulation of T Cell Motility, Chemotaxis, and Trafficking in the Lymph Node Paracortex

**DOI:** 10.1371/journal.pone.0045258

**Published:** 2012-09-19

**Authors:** Gib Bogle, P. Rod Dunbar

**Affiliations:** 1 Maurice Wilkins Centre, University of Auckland, Aukland, New Zealand; 2 Auckland Bioengineering Institute, University of Auckland, Aukland, New Zealand; 3 School of Biological Sciences, University of Auckland, Aukland, New Zealand; Whitehead Institute, United States of America

## Abstract

Agent-based simulation is a powerful method for investigating the complex interplay of the processes occurring in a lymph node during an adaptive immune response. We have previously established an agent-based modeling framework for the interactions between T cells and dendritic cells within the paracortex of lymph nodes. This model simulates in three dimensions the “random-walk” T cell motility observed *in vivo*, so that cells interact in space and time as they process signals and commit to action such as proliferation. On-lattice treatment of cell motility allows large numbers of densely packed cells to be simulated, so that the low frequency of T cells capable of responding to a single antigen can be dealt with realistically. In this paper we build on this model by incorporating new numerical methods to address the crucial processes of T cell ingress and egress, and chemotaxis, within the lymph node. These methods enable simulation of the dramatic expansion and contraction of the T cell population in the lymph node paracortex during an immune response. They also provide a novel probabilistic method to simulate chemotaxis that will be generally useful in simulating other biological processes in which chemotaxis is an important feature.

## Introduction

We have previously developed an agent-based model to simulate lymphocyte behaviour in the lymph node (LN), in which cells move on a three-dimensional lattice [1], [2], [3]. At its current stage of development, the model is restricted to processes involving T cells and dendritic cells (DCs) in the lymph node paracortex, and the aim is to represent T cell activation and proliferation during an immune response. This paper extends the model to include more detail on the processes influencing T cell migration and trafficking.

For any given pathogen there is only a very small fraction of T cells capable of recognizing and responding to the antigen (peptide fragments) presented by DCs in the paracortex, creating a kind of “needle in the haystack” problem for these very rare antigen-specific T cells to encounter their cognate antigen, become activated and proliferate. There is now ample evidence from intravital microscopy that the fundamental mechanism by which cognate T cells meet up with DCs in the paracortex is their apparent “random-walk” motility [4], [5], [6], [7]. On entering the paracortex from the blood, via high endothelial venules (HEV), T cells become highly mobile, using the random network of fibroreticular cells to roam throughout the region where the DCs are stationed [8], [9], [10]. In the relatively-short intravital tracking periods (typically no more than an hour) the cells appear to follow trajectories that are well described as persistent random walks, and it was initially thought that their motion was purely random (with characteristics strongly influenced by the random structure of the underlying fiber network). However some experimental observations suggest that subtle biases in the motion resulting from chemotactic signals, for example from activated DCs bearing antigen, could have significant effects on the immune response, even if those signals were below the level of detection in the early *in vivo* microscopy studies [8], [9]. As a first approximation we treated T cell motion as a persistent random walk, with parameters that have been determined experimentally – a mean speed of about 8–16 µm.min^−1^, and a coefficient of motility (analogous to a diffusion coefficient) of 50–100 µm^2^.min^−1^ [11], [12], [13], [14], [15], [16]. Nevertheless we have been interested to explore methods to incorporate subtle chemotactic effects into our model.

T cell chemotaxis has been hypothesized to influence T cell behavior in the lymph node in at least three contexts: in cell egress from the lymph node, in cell encounters with DCs, and in the migration of helper T cells to the B cell follicle. The investigations reported here concern the first topic. With the advent of *in vivo* multi-photon microscopy, T cell egress has become an active and much-debated research topic [17], [18], [19], [20], [21], [22], [23], [24], [25], [26], [27]. After stimulation with antigen, a T cell upregulates a surface molecule (CD69) [28], [29]. Some data suggest that this can suppress its response to a factor thought to allow T cells to leave the LN (S1P, acting through its receptor, S1PR1) [25], [30]. This may provide a mechanism for the temporary retention of activated cells in the LN, especially since another receptor (CCR7) responds to chemoattractants (CCL21/19) within the LN. It has been hypothesized that a “tug-of-war” takes place between these opposing forces at the exit portal [18], [31], determining the likelihood of egress.

There is evidence to suggest that chemotaxis could be a factor in the migration behaviour of activated cells. We have carried out adoptive transfer experiments to explore the factors influencing the size and timing of the immune response of CD8 cells (unpublished data). One striking observation from these experiments was that activated CD8 cells after initially being retained in the lymph node then leave at an accelerated rate starting about three days after antigen encounter. Initial retention of the activated cells is consistent with the upregulation of CD69 on exposure to antigen. While it is known that CD69 expression is downregulated after a couple of days [32], allowing S1PR1 expression to rise, and it has been suggested that this gives the cells permission to leave, our experimental results suggest that the activated cells are not simply given ability to leave, but leave at more than the expected rate, i.e. more rapidly than non-cognate cells leave under normal conditions. Such an enhanced egress rate could result either from an enhanced ability to exit when in the neighbourhood of an exit portal, or from an enhanced rate of arrival at a portal. One possibility is that the accelerated rate of egress of activated cells results from chemotactic attraction to the exit portals.

While the role of chemotaxis in T cell trafficking is not yet fully resolved, there seems to be little doubt that as more is learned about T cell behaviour in the lymph node chemotactic influence will be found to be an important component. In the case of B cell behaviour in the follicle the essential function of chemotaxis in attracting activated BCL6+ cells to the germinal center and retaining them there is already clear. It is these considerations that have motivated the development of a method to handle chemotaxis within a model that simulates lymphocyte motility on a lattice. The function of modeling in this context is to aid in interpretation of the experimental results, and to provide a way of assessing hypotheses about possible mechanisms by comparing the results of simulations that incorporate them. An agent-based model able to simulate T cell trafficking, motility and chemotaxis in a 3D domain, accommodating a changing T cell population, and also capable of incorporating cytokine diffusion, is the appropriate tool for exploration of theories about the roles of chemokines and receptor expression in cell egress.

While T cell motility is the principal determinant of the rates of encounter of T cells and DCs, a related process that is of equal importance for the size of the immune response is T cell trafficking though the lymph node, in particular the dramatic changes in trafficking rates that occur during the event. In conditions of normal surveillance, with no infection, an individual T cell may transit through a lymph node in a time ranging from a few hours to a few days. The mean transit time, also known as the residence time, has been variously estimated to fall in the range 12–24 hours [33], [34], [35], [36]. Under steady-state conditions the residence time is inversely related to the trafficking rate; it is equal to the T cell population of the paracortex divided by the inflow rate (which is the same as the outflow rate). During an immune response the rate of inflow of lymphocytes into a draining lymph node increases many-fold, and the resulting imbalance between inflow and outflow causes the T cell population of the paracortex to grow by a factor of 5–10 at the peak [37], [38]. As the infection is cleared the population declines to the steady state level again. Clearly this preferential location of T cells within a lymph node where there are DCs carrying evidence of the invading pathogen increases the likelihood of activation of the rare cognate T cells, speeding up the body’s defensive response. Given the crucial significance of trafficking in the immune response, it is an important component of a comprehensive model of T cell activation.

Models of T cell behavior within a lymph node are handicapped by the lack of a good description of the layout of the region where the cells move, in particular of the number and locations of both the inlet (HEV) and the egress portals. There is a patchwork of information that has been acquired in several different ways: histology [39], [40], [41], [42], [43], scanning electron microscopy [44], [45], and explant and *in vivo* confocal microscopy [8], [9], [13], [15], [46], [47], [48], [49], [50], [51], [52], [53]. Where images are highly detailed they are of limited extent, and usually 2D. *In vivo* microscopy has yielded (among other things) a wealth of data concerning T cell motility, and the nature of T cell-DC and T cell-B cell interactions, but so far it has not shed much light on T cell egress. A recent study [19] that employed confocal microscopy on serial sections achieved 3D reconstruction of part of a lymph node, providing new information about the organization of the cortical sinus network, and suggesting that egress occurs via blunt-ended sinuses. However given the gaps in current knowledge of the 3D topography of ingress and egress points, it seems acceptable to model the sites of these points in a simplified manner, where all assumptions are clearly identified, and where possible, the sensitivity of the model predictions to input parameters is explored.

In this paper we report how our agent-based T cell model has been extended to include both a new treatment of the spatial relationship between ingress and egress points into the paracortex, and a new method for simulating chemotactic effects. The extended model has been used to explore how a chemotactic influence on egress might play a role in an adaptive immune response. Besides the incorporation of chemotaxis into the model for cell motion on a lattice, model modifications include an improved treatment of the way T cell influx is modulated by growth factor signals during an immune response, and a method for automatically varying the number and location of exit portals as the T cell population grows and declines. Although the main aim of this work has been to investigate the simulation of chemotaxis, careful attention has also been given to the other processes affecting T cell trafficking, because it is necessary to ensure that together they generate reasonable system behavior, in particular maintenance of steady-state T cell populations under normal conditions. We present the methods used, and numerical experiments investigating the implications for T cell trafficking. Simulation results are mostly presented in statistical form, through the probability distribution of transit time and its average, the residence time. It is shown that if a subpopulation of T cells is susceptible to egress chemotaxis, the residence time of these cells is significantly reduced. While this result is not a surprise, the modeling drew attention to the fact that it is the relative level of chemotactic influence that is important.

## Methods

### The Foundation of the Model

The simulation model deals with processes within the dense paracortex, the region of the LN that is packed with T cells and where DCs bearing antigen are stationed. Details of the basic model framework, including cell motility and the variation in cellular population, have been published previously [1] therefore only a summary will be provided here. T cell motion is simulated on a 3D lattice, in 15 second time steps, as a persistent random walk, in a way that can reproduce the main parameters of cell motion as measured by intravital microscopy. DCs in the model do not move since their motility observed *in vivo* is trivial compared to that of T cells. The lattice grid spacing is determined from the amount of paracortical volume taken up by the T cells – the simulations reported here assume that the cells occupy 60% of the volume, giving a grid spacing of 6.3 µm (the rest of the space being taken up by other cell types, fibres and fluid). Cells occupy a roughly spherical region (referred to as the “blob”) within the cubic lattice. The blob grows then contracts during an immune response, with the number of lattice sites available for cell occupancy always matching the number of cells, where a T cell occupies a single site and the soma of a DC takes seven sites. When the number of cells changes the availability of sites is changed to maintain the equality – entry of a T cell, or cell division causes another site at the boundary of the blob to be made available for a cell to occupy, and cell egress or death causes an available site at the boundary to be made unavailable. Interactions between T cells and DCs are probabilistic, and can occur when a T cell is with a DC’s sphere of influence (the region spanned by its dendrites). Non-cognate interactions last for up to 10 min, with a median contact time of 3 min [7].

One of the main features of this method is that it accounts for cell crowding, while benefitting from the computational efficiency of on-lattice motion. Modelling approaches that account for the dynamics of cell-cell collisions are extremely demanding computationally, and not attractive when cell populations on the order of 10^5^ must be simulated. On the other hand, models that treat the motion of each cell as being independent of that of all the other cells, implicitly allowing cells to occupy the same space, cannot prevent the occurrence of unphysical concentrations of cells. This is a particularly serious issue when there is chemotaxis – all cells subject to the chemotactic influence will gravitate to the same region. The problem of simulating motion on a packed lattice is addressed by allowing two cells to occupy the same lattice site for a single time step, enabling passing when cells meet. This is an effective compromise that limits crowding, even in the presence of chemotaxis, while permitting large cell populations to be handled efficiently.

The inevitable simplifications inherent in such a model are acceptable provided the following conditions are met: (1) the simulations must reproduce the experimentally observed residence time for T cells in the paracortex, (2) the results should not vary significantly when the scale of the simulation is varied (initial population of the blob), and (3) on the winding down of the immune response, the steady-state condition should be restored.

### Definition of Sites for T Cell Ingress and Egress

**Figure 1:**
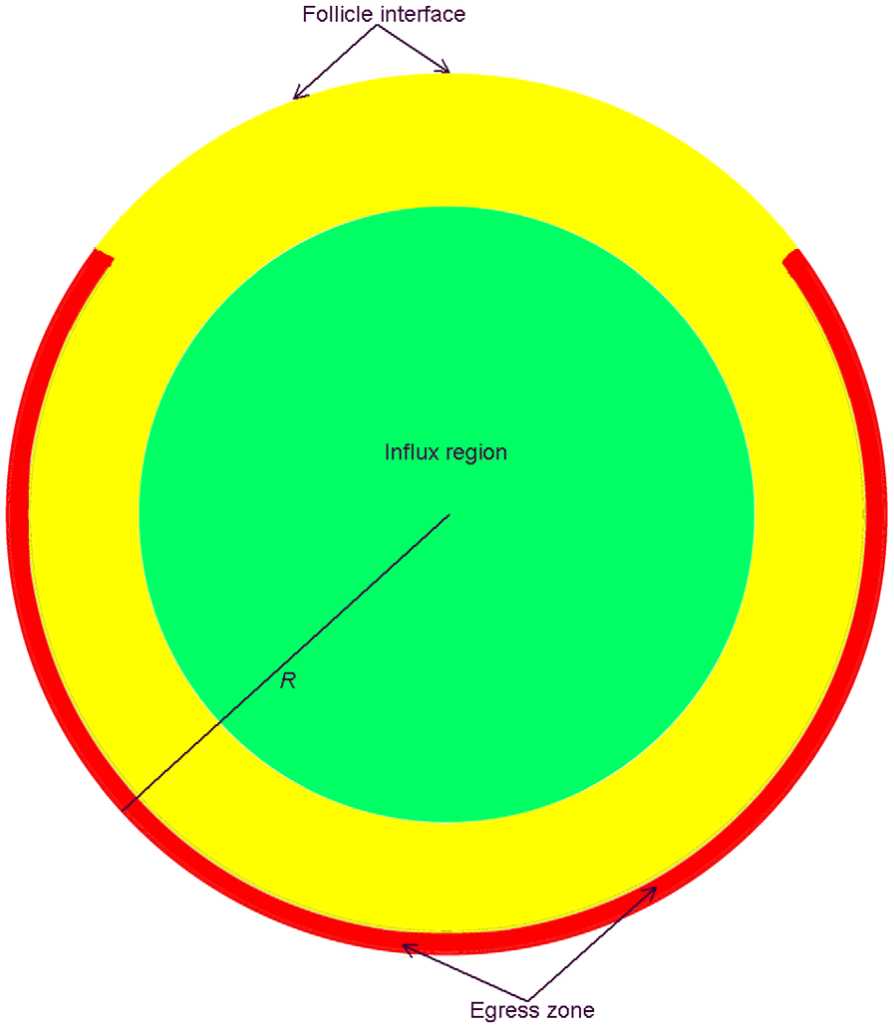
Regions of the spherical “blob” representing the paracortical T zone. T cells enter at random locations within a sphere of radius 0.7 *R* (green). Exit portals are located within a layer near the boundary (red), except for a “cap” representing the interface with the B cell follicle.

In the earlier version of the model T cells entered and left the blob at random locations distributed throughout the volume, reflecting uncertainty about the actual layout of the paracortex. The three-dimensional structure of the lymph node as a whole, and the dense paracortex in particular, is still poorly characterised, but we do know that it is highly complex and variable. There is still some debate about how and where T cells leave the paracortex [18]. While lymphocyte egress through portals into the medullary sinuses has been observed [23], some recent evidence suggests that blunt-ended cortical sinuses may be an important route for exiting cells [19]. Both because the necessary descriptions of LN structure and T cell behaviour are not available, and because of the enormous computational requirements of a more realistic simulation, we have now opted for an intermediate level of detail in which cell exit occurs near the boundary, and entry occurs within the blob interior (Fig. 1). In order to limit the ability of cells to leave the blob shortly after entry, entry locations are restricted to lie within a sphere with radius 0.7 of the blob radius (Fig. 1). In introducing these new conditions regarding T cell ingress and egress, we have tested whether they still allow the model to meet the fundamental biological requirements mentioned above.

### The Number of Exit Portals, and their Permissiveness to Lymphocyte Egress

The model assumes that T cells leave the paracortex at discrete locations, or portals [26]. These egress locations are randomly distributed near the blob boundary, but excluding a cap-shaped portion (defined by *x*/*R* >0.6, where the blob center is the origin of the axes and the radius is *R*) intended to represent roughly the interface with a B cell follicle (see Fig. 1). A cell at any site in the Moore neighborhood of a site defined as an exit portal location has a possibility of egress. (The Moore neighborhood is the block of 27 sites centered on the site.) The residence time *T_res_* is determined by the T cell population *N*, the number of exit portals, *N_E_*, and the probability of egress of a cell in the neighbourhood of a portal, *P_E_*. Of these three parameters the value of *T_res_* for normal steady-state conditions is known approximately (12–24 h) but currently even rough estimates of *N_E_* and *P_E_* are not available. The approach we have tentatively adopted is to fix the exit probability *P_E_*, then through simulations determine the dependence of *N_E_* on *T_res_* and *N*. These simulations were carried out with a constant T cell influx rate (i.e. no inflammation) set equal to the initial T cell population divided by *T_res_*. When the cell population is maintained at the initial level we can be sure that the model is reproducing the specified residence time. (These calibration simulations were carried out in the absence of DCs.) The probability of egress within one 15 second time step for a cell in this neighborhood has been arbitrarily fixed at *P_E_* = 0.02.

The function relationship between the number of exits and the T cell population was determined empirically through extensive simulations. The procedure for checking that the residence time is reproduced is to run a simulation with a constant T cell influx rate (i.e. no inflammation) set equal to the initial T cell population divided by *T_res_*. When the cell population is maintained at the initial level we can be sure that the model is reproducing the specified residence time. (These calibration simulations were carried out in the absence of DCs.).

### Inflammation, Vascularity and Cell Ingress

The rate of lymphocyte influx rate into a LN varies widely during an adaptive immune response, and we thought it important to build such changes into our model. The dramatic expansion in LN size in the presence of infection, or other inflammatory stimuli, is largely the result of increased recruitment of lymphocytes to LNs. An increase in blood flow into a reactive LN [38], [54] increases the rate at which lymphocytes pass through the LN, though it is not clear what drives this increase in blood flow. Proposed mechanisms include the redistribution and straightening of existing vessels [54], vascular dilation and the growth of new vessels [38]. From experiments using highly-detailed vascular casting, Steeber et al. concluded that in response to antigen stimulation there is an initial period of vascular dilation and redistribution followed by vascular proliferation [55]. Inflammation signals in the form of TLR agonists have been shown to drive remodeling of the primary feed arteriole, increasing its flow capacity [56], either directly though binding to the vascular endothelial cells or indirectly by stimulating DCs to secrete cytokines that act on the endothelial cells. Endothelial cell expansion has been attributed to VEGF secreted by activated DCs [57], and the same researchers also showed that fibroreticular stromal cells are the main VEGF-producing cells in the LN, implying that these cells have a major role in regulating LN vasculature [58]. We have therefore developed a sub-model of LN vascular remodeling that is driven by factors such as VEGF; this sub-model can be refined as the processes controlling vascular changes in reactive LNs are better understood.

It is assumed that an inflammation signal from the site of infection, presumably carried to the LN by the lymphatic flow, drives the changes in T cell influx. This signal stimulates the production of growth factors such as VEGF, for example by stromal cells and DCs, which in turn influence vascularity. The word “vascularity” is not given a precise meaning – whether there is indeed growth of new HEV capillaries, or whether the existing capillaries expand or become more permeable to T cells is not determined – but it is assumed that the rate of passage of T cells into the paracortex is in direct proportion to this vascularity value. There is a basal level of growth factor production, which maintains vascularity at its steady-state level ( = 1) in the absence of inflammation.

For convenience, let *G* represent the growth factors. The amount of *G* at time *t*, in arbitrary units (say growth factor units, gfu) is *M_G_(t)* and the number of T cells in the blob is *N(t)*. The fluid volume is assumed to be proportional to *N(t)*, therefore the concentration of *G* (in units of gfu.cell^−1^) *C_G_(t)* is:
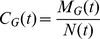



The rate of *G* production is made up of a baseline rate, proportional to the initial blob size (equivalently to the initial T cell population), and an additional rate proportional to the product of the initial blob size and the current inflammation signal, *A(t)*, which is normalized to the range (0,1). *G* is also subject to decay at a rate *δ_G_*:
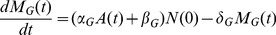
(1)


Relative vascularity *V(t)* grows at a rate proportional to the product of *V(t)* and a saturating function of the *G* concentration, described by a Hill function *H*, while also decaying at a rate *δ_V_*:
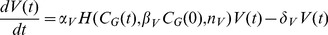
(2)where 
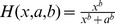


The relative vascularity determines the T cell influx rate *F_in_(t)* by scaling from the steady-state influx rate *F_in_(*0*)*: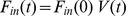, where 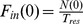. At equilibrium, with no inflammation signal, the amount of *G* is constant, and from Eq. (1) is given by 
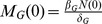
, therefore the equilibrium concentration of *G* is 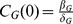. From Eq. (2) we can express *δ_V_* in terms of the other parameters:
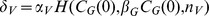



The simulations reported in this paper have been carried out with free parameter values as listed in Table 1. A simulation run start with vascularity at the steady-state level, *V(0)* = 1, and the level of inflammation (with a maximum value of 1) is specified in advance as a function of time. Typically a simple time course is specified, with inflammation held at a fixed value for some period, then tapering to zero.

**Table 1:** Parameter values for the inflammation-influx sub-model.

Parameter	Value
*α_G_*	4.0 E−7 gfu.cell^−1^.min^−1^
*β_G_*	5.0 E−8 gfu.cell^−1^.min^−1^
*δ_G_*	0.002 min^−1^
*α_V_*	0.001 min^−1^
*β_V_*	2.0
*n_V_*	2.0

### Chemotaxis Sub-model

We developed a general approach to incorporating a chemotactic influence into the probabilistic simulation of motion on a lattice. This approach aimed to allow cells moving within the lattice to “sense” chemotactic gradients in 3D space – including multiple gradients emanating from different sources within the lattice – and alter their motility in a probabilistic manner, depending on the relative strength of those chemotactic gradients. A key element of the chemotaxis model is that T cells moving within the lattice display a *tendency* to move up chemotactic gradients, while maintaining the random walk motility pattern observed in all intravital microscopy experiments to date (referred to below as “normal motility”).

The mechanisms that determine a cell’s response to chemokines are complex, but it is generally accepted that in the case of chemotactic attraction a cell tends to move up the chemokine concentration gradient, and the degree of chemotactic influence on the motion increases with increasing concentration gradient. The obvious first step for simulating chemotaxis, then, is to simulate the chemokine concentration field. While the approach described below is directly applicable to situations where chemokine concentrations are simulated, including multiple chemokines, it is also amenable to a convenient simplification in the case of egress via discrete exit portals, employing the mechanism of control of cell egress by the gradient of some chemokine close to an exit portal [18], [31]. In this case, instead of solving for chemokine concentrations it is reasonable to use a simplifying assumption to approximate the chemotactic influence, as a function of distance from the source of the chemokine – in the case simulated, the exit portal.

Consider a T cell that is experiencing a chemotactic attraction represented by ***C***, where ***C*** is a 3-vector. The strength of the attraction is given by |***C***|, and it is in the direction given by the unit vector ***v*** = ***C***/|***C***|. Note that the strength of the chemotactic attraction is not given physical units; rather it is a quantity that can be translated into an effect on the probabilities that govern the cell’s motion on the lattice. In the absence of chemotaxis the jump probabilities associated with the *N_J_* possible jumps to neighbour sites (e.g. *N_J_* = 26 for the Moore neighbourhood) are computed in the usual way [1]. (Note that all distances are expressed in lattice grid units, therefore a site’s near neighbours are at a distance of 1, √2 or √3.) This calculation yields the set of jump probabilities {*p*(*i*), *i* = 0,.., *N_J_* }, where *p*(0) is the probability of no jump. A set of normalized jump probabilities {*p_c_*(*i*), *i* = 1,.., *N_J_* } is computed as follows to represent the directional effect of chemotaxis alone at the specified cell site, without reference to its strength. For each possible jump direction, the probability is made proportional to the square of the cosine of the angle between the jump vector and the unit vector ***v***, scaled by the inverse of the jump distance, and setting to zero the probabilities corresponding to jumps directed counter to the attracting influence, i.e. those for which the cosine is negative. Dividing by the jump distance is necessary because there are three possible values.

**Figure 2:**
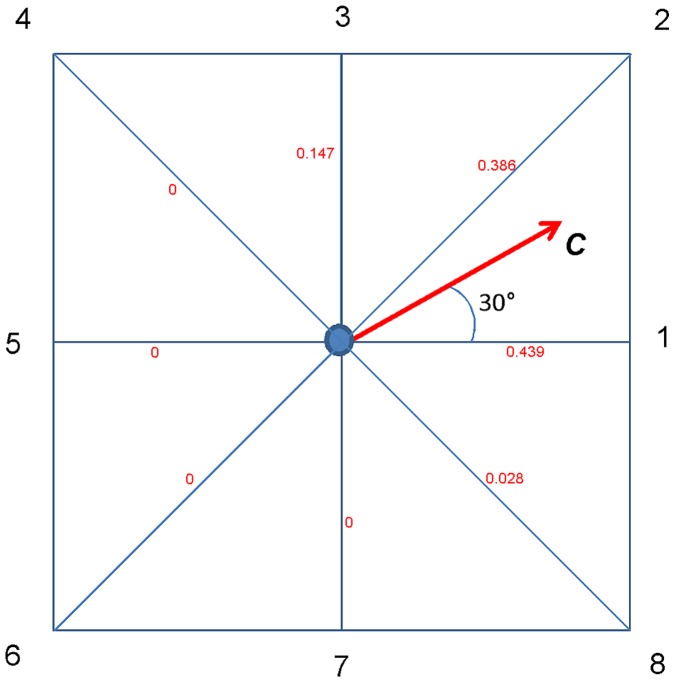
Chemotactic jump probabilities on a 2D lattice. In the simplified 2D case there are eight possible jump directions. For illustration purposes the jump probabilities have been computed for the example of a chemotactic attraction vector oriented at 30° from the x-axis, i.e. ***C*** = (0.866, 0.5). The figure indicates for each jump direction, *i* = 1,.8, the associated chemotaxis-only probability *p_c_*(*i*) of the jump (see Table 2).

For each *i* = 1,., *N_J_*, let ***u***(*i*) be the vector representing a jump in the *i*th direction, then the angle between ***v*** and ***u***(*i*) is *θ_i_*, where 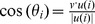. The relative amount of chemotactic influence in the direction ***u***(*i*) is given by *w*(*i*), where 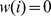 if cos(*θ_i_*) <0, else 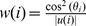. Then for each *i* = 1,..,*N*, the probability *p_c_*(*i*) is proportional to *w*(*i*):
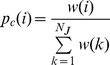



The situation in 3D with 26 jump directions is not easily conveyed by a diagram, but it is readily visualized in the case of motion on a 2D lattice, for which *N_J_* = 8. In the example illustrated in Fig. 2 the chemotactic attraction vector ***C*** has direction (0.866, 0.5), i.e. it is oriented 30° from the direction of the jump to neighbour site 1. Table 2 shows the steps in computing the chemotaxis-only jump probabilities *p_c_*. which sum to 1, determine the relative contributions of the different possible jump directions to chemotaxis-only motion at the site. The relative amounts of chemotaxis and normal motility depend on *|* ***C*** *|,* the strength of the chemotactic influence, which takes into account the susceptibility of the T cell to chemotaxis. This susceptibility may in general vary between cells, and between states of differentiation of a single cell. If the chemokine gradient vector is ***G***, then we might set ***C*** = *K_E_* ***G***, where *K_E_* is a parameter representing the cell’s chemotactic susceptibility. Then (assuming appropriate scaling) the fractional influence of chemotaxis is given by *α*, taking values in the range (0,1): 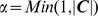. The jump probabilities modified to account for chemotaxis, *p* ^*^, are computed as follows. The probability of no jump is 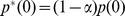, while for *i* >0 it is necessary to take into account the possibility that some jump directions are prevented by cell crowding: if 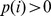 then 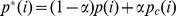, else 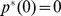.

**Table 2:** Calculation of chemotactic jump probabilities *p_c_* for the 2D-lattice example of Fig. 2.

*i*	*θ_i_*	cos(*θ_i_*)	|*u*(*i*)|	*w*(*i*)	*p_c_*(*i*)
1	30°	0.866	1	0.75	0.439
2	15°	0.966	1.414	0.66	0.386
3	60°	0.500	1	0.25	0.147
4	105°	−0.259	1.414	0	0
5	150°	−0.866	1	0	0
6	165°	−0.966	1.414	0	0
7	120°	−0.500	1	0	0
8	75°	0.259	1.414	0.047	0.028

The chemotactic attraction vector ***C*** is oriented 30° from ***u***(1). The cosines of the angles between ***C*** and the jump vectors for neighbour sites 4, 5, 6 and 7 are all negative, therefore probabilities of jumps in these directions are zero. Note that the probability of jump ***u***(1) is slightly greater than that of jump ***u***(2), even though ***C*** is closer to ***u***(2) than to ***u***(1). This is because jump ***u***(2) has length √2 = 1.414, compared with 1.0 for ***u***(1), therefore when weighted by the length, the expected jump in direction 2 exceeds that for direction 1.

The assumption that the model uses to treat attraction to exit portals is to make the influence of an exit depend simply on the distance of the cell from the exit, *r*, through some function *g*(*r*) with a maximum value of 1. A simple choice for *g*(*r*), to convey how the chemotactic effect falls off with distance from the exit, is a bounded inverse square relation: 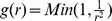. In this case the vector ***v*** that conveys the direction of the attraction, and is used to compute the *p_c_* values, is the unit vector corresponding to the relative offset of the exit portal from the cell’s location. The fractional influence of chemotaxis is given by 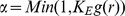.

When a cell is subject to more than one chemotactic influence, e.g. from multiple chemokines, or, in the T cell model, if it is within the range of influence of multiple exit portals, vector addition offers a straightforward procedure to combine the influences. Consider a cell subject to influence from *M* chemokines, manifested as chemotactic attraction vectors ***C*** *_j_*, *j* = 1,..,*M*. The net chemotactic attraction vector can be defined to be the vector sum of the *M* influences,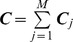. The jump probabilities can then be computed in the usual way, using direction unit vector ***v*** = ***C***/|***C***| to compute *p_c_* and strength 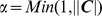 to combine *p* and *p_c_* to give *p* ^*^. In the case of a cell that is within the range of attraction of more than one exit portal the combined effect of the multiple influences can be computed in the same way. Consider *M* attracting sites, with direction offsets from the T cell location conveyed by unit vectors ***v*** *_j_*, *j* = 1,..,*M*, and distances given by *r_j_*. The strength of each individual chemotactic attraction is given by 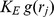, and the net chemotactic influence vector is 
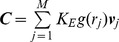
.

### Transit Time

Under steady-state conditions the mean T cell transit time, the residence time *T_res_*, is related to the number of cells in the blob and the inflow rate *F_in_* (which is also the outflow rate) through the equation *T_res_* = *N*/*F_in_*. Transit times of individual cells can vary significantly about this mean. The probability distribution of transit time is computed by simulating a steady-state condition, then recording both the entry and exit times of a large number of cells as they traffic through the blob. The transit time distribution has been determined for the cases in which all cells experience the same influence of exit chemotaxis, and for some cases in which a variable fraction of cells are subject to chemotaxis.

## Results

### Number of Exit Portals

For a given value of egress probability *P_E_* the total rate of cell egress is expected to vary in proportion to the number of exit portals. Since the cell flux at steady-state must equal the number of T cells in the blob (*N*) divided by the residence time (*T_res_*), the number of exit portals *N_E_* needed to maintain steady-state can be expected to be approximately proportional to *N*/*T_res_*.

**Figure 3:**
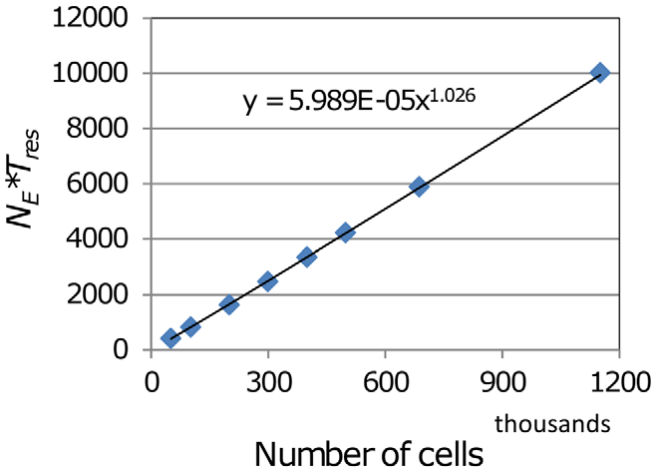
Fit of a power function to the product of *N_E_* and *T_res_*. Simulations were conducted with T cell population *N* ranging from 51 k to 1150 k and influx rates corresponding to a residence time of 12 h, to determine in each case the number of exit portals *N_E_* required for maintenance of steady state. The product (number of exit portals)x(residence time) was plotted against *N*, and the best-fit power function determined. Further simulations confirmed that the number of exits predicted by this function also achieve steady-state system behaviour for cases with a residence time of 24 h.

Initial simulations were carried out with *P_E_* arbitrarily set to 0.02. Through repeated simulation runs the value of *N_E_* required to maintain steady state was determined by iterative adjustment, for initial T cell population *N* ranging from 50 k to 1.1 M. A nearly linear power law was then found to provide a good fit for the relationship between *N* and *N_E_* (Fig. 3):
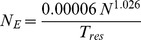
(3)


This formulation satisfies requirements (1) and (2) specified above. For any choice of *T_res_* consistent with observations (i.e. in the range 12–24 hours), simulations with T cell populations ranging from 50 k to 1.1 M reproduce the specified residence time. Note that the egress probability *P_E_* of 0.02 effectively determines the number of exits, since a larger (smaller) value of *P_E_* would require a smaller (larger) value of *N_E_* to generate the rate of egress needed to reproduce the desired residence time. It is to be expected that *P_E_* and *N_E_* will be in an inverse relationship to each other, since the total number of exit neighbour sites is proportional to *N_E_*, while the rate of egress is proportional to the product of *P_E_* and the number of exit sites. This expectation was confirmed by simulations with different values for the exit probability parameter: in runs with *P_E_* = 0.01, setting the number of exits to twice the value generated by Eq. (3) maintains steady-state, and with *P_E_* = 0.005 a multiple of four is required to keep the T cell population constant. This led to the more general expression for the number of exits:
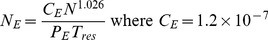
(4)


Hence extensive simulations showed that the functional relationship between the number of exits *N_E_* and the T cell population *N* was found to be almost linear. Effectively, the strategy of introducing a fixed number of exit portals, randomly distributed throughout the designated exit zone (Fig. 1), worked well in generating steady state T cell flux, regardless of the starting T cell population. As the starting T cell population increased, the number of exits required to generate a desired T cell residence time rose proportionally, as more T cells needed to “find” exits with a fixed permissiveness to their egress. Should exit portals change their permissiveness to T cell egress, the number of exits needed to support steady state flux falls inversely.

### Inflammation

The results reported above are for steady-state conditions, in which the influx rate is fixed and the T cell population remains approximately constant. In the case of an immune response, inflammation signals cause the T cell population to grow, and when the inflammation dies away the population falls. As the cell population changes the model automatically adjusts the number of exit portals, ensuring that Eq. (3) is always satisfied. The locations of the exit portals are also adjusted automatically as the blob expands and contracts, in a way that keeps them near the boundary (such that the 27 sites of the Moore neighbourhood of an exit portal are all within the blob, and at least one of these sites has a neighbour site that is outside) and also maintains their separation. To focus on the effect of inflammation and check that the growth factor model performs correctly in conjunction with the way the number of exit portals is determined, simulations were run with specified time variation in the inflammation signal, but no corresponding antigen influx and no DCs. The resulting variation in the T cell population over the 10-day course of the simulation is therefore solely the result of trafficking changes. For these runs the initial population was 100 k, the equilibrium residence time was either 12 or 24 hours, and the parameters of the trafficking sub-model were given the values listed in Table 1. In each case the inflammation signal *A*(*t*) was held at a constant level for 3.5 days then ramped to zero over the next 24 hours.

**Figure 4:**
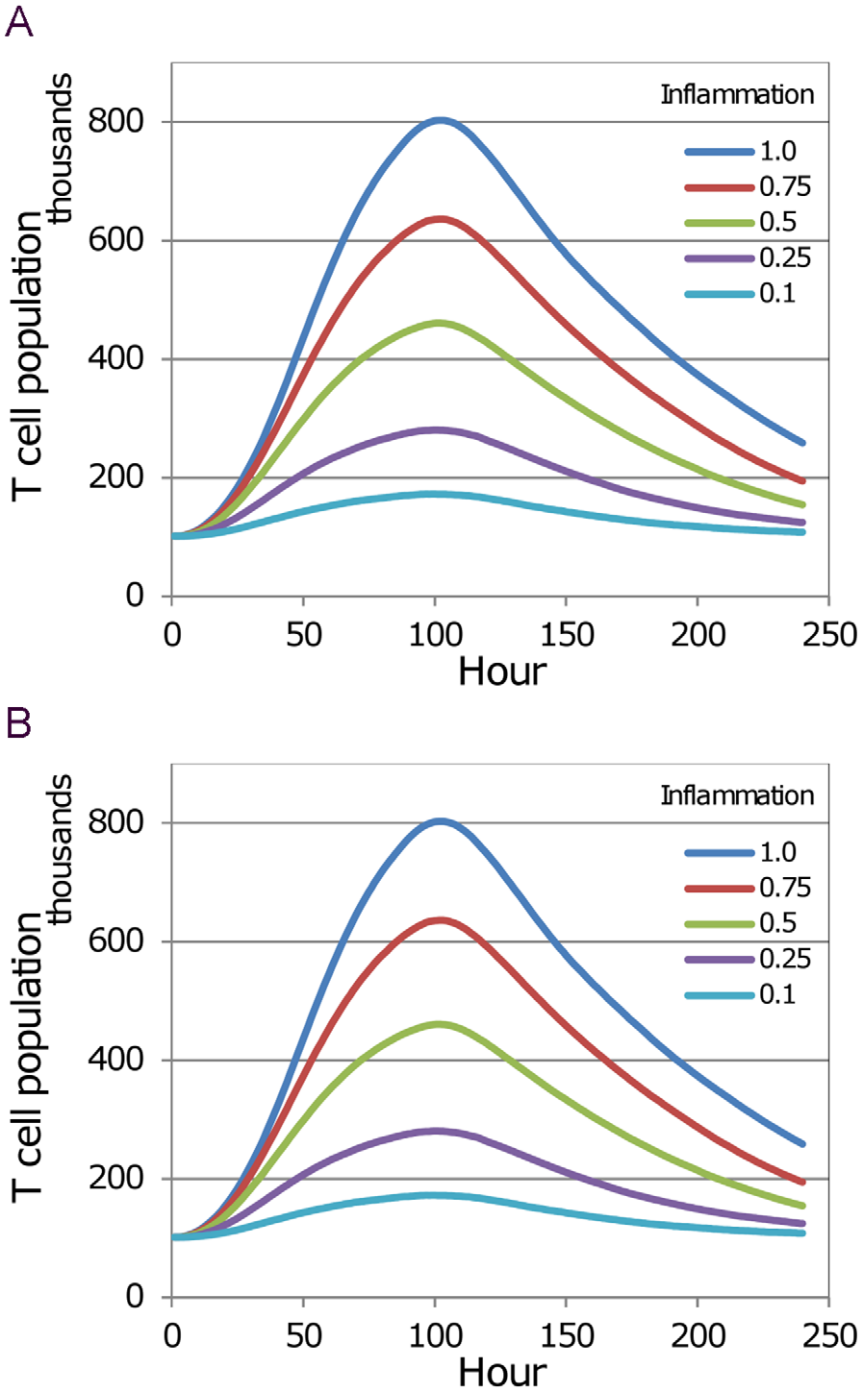
Time course of simulated T cell population for varying levels of inflammation signal. In each 10 day simulation run, with a starting population of 100 k T cells, the external inflammation signal was held at fixed level (0.1, 0.25, 0.5, 0.75, 1.0) for 3.5 days, then ramped to zero over 24 hours. The T cell population of the blob rises initially while inflammation-driven influx exceeds efflux, then falls back towards the initial level as steady-state balance of inflow and outflow is restored. (A) Simulations with *T_res_* = 12 h. (B) Simulations with *T_res_* = 24 h.

The five cases plotted in Fig. 4, corresponding to *T_res_* = 12 h and 24 h, are for inflammation levels of 0.1, 0.25, 0.5, 0.75 and 1.0. (These results have been replicated by using Matlab to solve the ODE system given in Supplementary Info, with the addition of this formula for the outflow rate: *F_out_* = *N*(*t*)/*T_res_*.).

These simulation results show that the trafficking model (i.e. the joint operation of the influx model and the egress model, including the specification of the number of exit portals as a function of the number of T cells) satisfies the third requirement that we specified: after rising in response to inflammation, the T cell population returns to the steady-state level when the inflammation dies away.

### Chemotaxis

**Figure 5:**
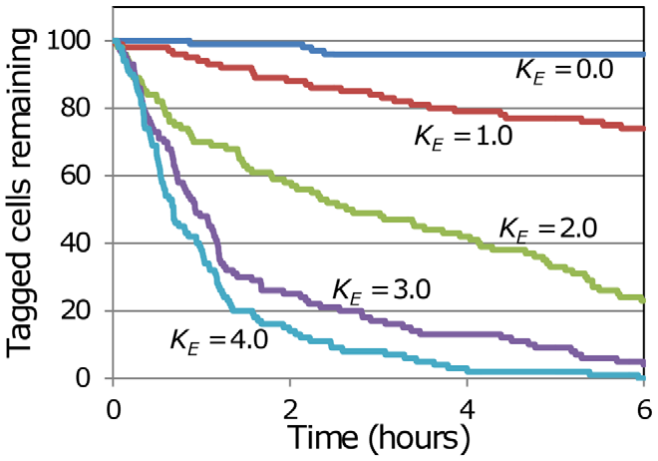
Cell retention with varying levels of egress chemotaxis. To explore the influence of chemotaxis in a simplified setting, simulations of steady-state trafficking were carried out for a blob of 10 k cells with a single exit portal at the centre. All cells were non-chemotactic, except for 100 tagged cells initially at randomly-selected locations within a sphere of radius = 7 grids centred on the exit portal. The trajectories of these chemotactic cells were recorded over six hours of simulation. For five simulation runs with a range of values of the chemotaxis parameter *K_E_*, the number of tagged chemotactic cells remaining in the blob has been plotted as a function of time.

Although the chemotaxis sub-model is generally applicable to chemotactic influences within the LN, for convenience we simulated the effects of a chemotactic influence emanating from the exit portals. This allows tracking of the effects of chemotaxis by tracking its effect on egress from the portals, which can be quantified through the residence time. We are not expressing acceptance of the reality of chemotactic egress, which is still undecided, but we do believe that the methods we have developed are appropriate for simulating this phenomenon. To provide a visual impression of how chemotaxis influences cell motion, a series of Supplementary Videos have been created and made available online as supporting material. In each case a simplified scenario was simulated to make interpretation easier, using the following procedure. Steady-state cell trafficking was simulated in a spherical blob with a radius of 13.5 sites, containing approximately 10 k T cells. There was a single exit portal located at the blob centre. The cell influx rate was adjusted to maintain steady-state, with the exit probability parameter *P_E_* = 0.02. In a simulation run, a specified number of cells that are initially located less than a specified distance *D* from the exit portal are randomly selected, made subject to a specified level of chemotaxis, and tagged. As the simulation proceeds the locations of the tagged cells are recorded. The videos display the motion of these tagged cells for four hours. Fig. 5 shows how the number of tagged cells remaining in the blob varies over the first six hours, for the cases with *D* = 7 grids (i.e. 44.1 um). In the absence of chemotaxis (*K_E_* = 0), the number of tagged cells in the blob declines slightly, as a result of the small probability (*P_E_ = *0.02) that cells adjacent to the exit portal will egress. As the chemotaxis influence increases, the tagged cells have a greater tendency to move towards the exit portal, so the number of tagged cells remaining in the blob declines more rapidly. However even should these tagged T cells find themselves adjacent to the exit portal, they still only have a small probability (*P_E_* = 0.02) of egressing within the next time step. Hence tagged cells can move “past” the exit portal and return to “explore” other sites within the blob. As the videos show, under this probabilistic method of simulating chemotaxis, the cells under chemotactic influence still maintain their random walk motility, so they have a *tendency* to move towards the exit portal, without moving directly up the chemotactic gradient. Importantly, this chemotactic tendency is occurring inside a swarm of other (untagged) cells, which are continuing their random walk motility without any chemotactic responsiveness. Hence the method allows chemotaxis to operate on sub-populations of cells in crowded environments that are swarming with cells undergoing random walk motility.

To further test the methodology, we simulated chemotactic influences within larger blobs with a full complement of exit portals. Two scenarios were simulated: in the first, all T cells were equally susceptible to chemotaxis; in the second, only a subset of T cells was subject to chemotaxis. This latter scenario is of interest because of abundant data showing that the expression of chemotactic receptors by lymphocytes changes dynamically during an immune response, so that at any stage of an immune response, different lymphocyte populations may differ substantially in their responses to a particular chemotactic influence.

**Figure 6:**
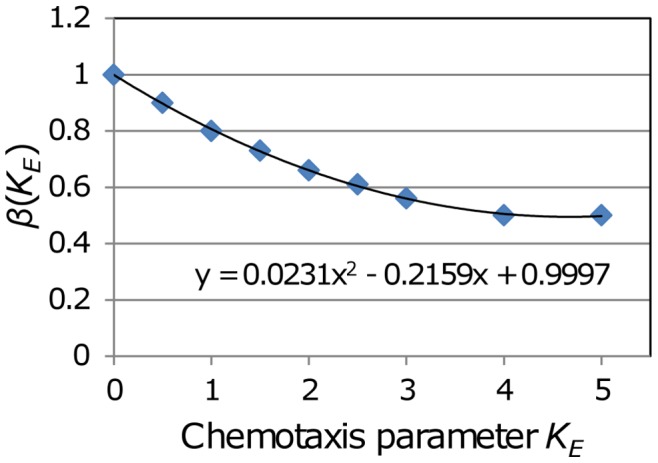
Adjustments to *N_E_* to maintain steady-state with varying *K_E_*. The formula for the base number of exit portals *N_E_* was determined with no chemotaxis (*K_E_* = 0). When there is exit chemotaxis (*K_E_* >0) the number of exits must be reduced to ensure that the steady-state T cell population is maintained. Numerical experiments were used to determine the needed reduction factor *β(K_E_)* for *K_E_* ranging up to 5.0.

Under the first scenario, where all T cells are sensitive to the chemotactic influence of the exit portals (*K_E_* >0), the T cells tend to crowd around them, with a concomitant increase in the rate of egress. Under these circumstances, to maintain steady state, the number of exit portals must be reduced. Simulations were therefore conducted to determine the reduction factor *β*(*K_E_*) that must multiply Eq. (4), for values of *K_E_* ranging from 0 to 5.0. The results, shown in Fig. 6, are approximately fitted by a quadratic function, *β*(*K_E_*) = 0.0231 *K_E_* ^2^ - 0.2159* K_E_* +0.9997. For *K_E_* greater than about 4.0 the *β* value required for steady-state stabilizes at 0.5, because at this high level of chemotaxis the exit portal neighbour sites are constantly fully occupied (with two cells per site), and further increases in chemotaxis have no effect. Simulations with the number of exits set to *β*(*K_E_*) times the value given by Eq. (4) maintained steady-state over the range of starting T cell populations from 50 k to 1.1 m, with residence times of both 12 and 24 hours. Incorporating *β*(*K_E_*) into Eq. (4) yields:
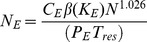



This expression provides the number of exit portals required in the model to maintain steady state, given the number of T cells, *N*, the residence time, *T_res_*, the egress probability, *P_E_*, and the chemotactic susceptibility, *K_E_*. Effectively these simulations show that chemotaxis acting on all cells is subject to rapid saturation, as spatial constraints reduce the ability of new cells to access the source of the chemotactic factors.

**Figure 7:**
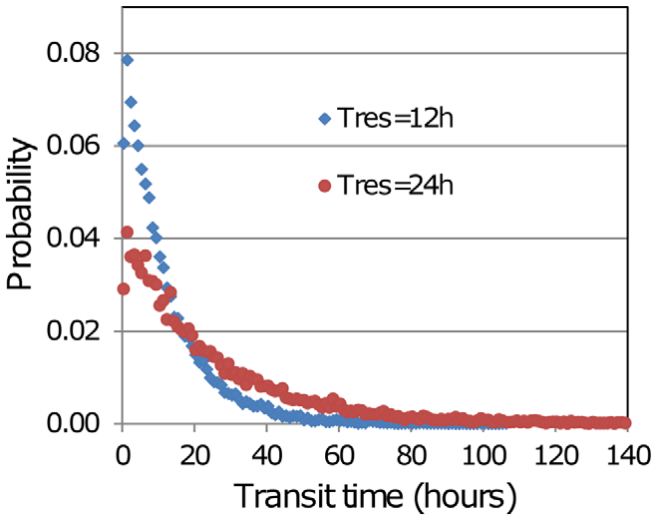
Probability distributions of transit time with no chemotaxis. Two steady-state simulations were carried out of with a T cell population of *N* = 50 k and an influx rate and exit portal count corresponding to a residence times of *T_res_* = 12 h and 24 h. 10 k cells were tagged on entry and their transit times were recorded on exit. The longest transit time (with *T_res_* = 24 h) was 205 h, but the graph has been truncated for clarity. (Note that in these simulations no time restriction was placed on the ability of cells to exit immediately after ingress.).

**Figure 8:**
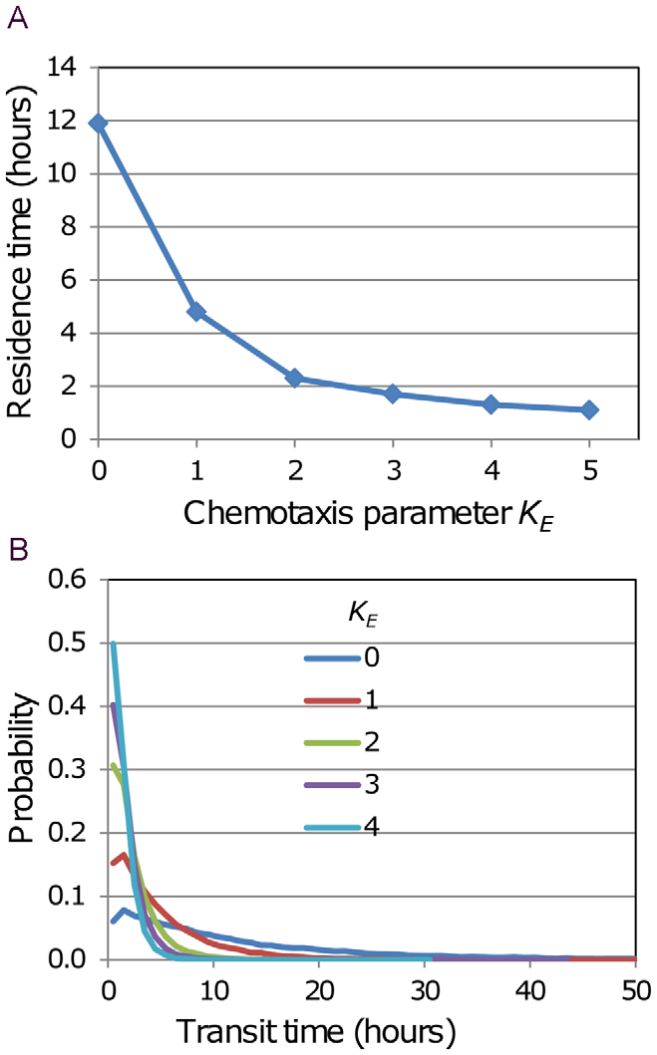
Dependence of transit time distribution on *K_E_* for cells subject to chemotaxis. In steady-state simulations with 50 k cells and with the number of exit portals and influx rate corresponding to *T_res_* = 12 h, 10% of the cells entering in the first 48 hours were tagged and made susceptible to chemotaxis, with values of *K_E_* ranging from 1.0 to 5.0. The tagged cells were tracked and their transit times determined. Results from the five simulations with *K_E_* >0 are compared with the no-chemotaxis case. (A) Dependence of residence time (mean transit time) on *K_E_*. (B) Transit time probability distributions (the distribution for *K_E_* = 5 is very close to that for *K_E_* = 4 and has been omitted for clarity).

Under the second scenario, only a subset of cells has enhanced chemotactic susceptibility, and the results are more interesting. In this case the more chemotactic cells might be expected to gain preferential access to the exit portals, and as a result leave the paracortex more rapidly. Hence we simulated this scenario, and measured the dependence of residence time on the chemotactic sensitivity (*K_E_*) of the susceptible subset. Probability distributions of transit times with varying levels of chemotaxis have been computed by keeping track of T cell entry and exit times. Steady-state conditions were simulated over 10 days for a T cell population of about 50 k using the influx rate corresponding to *T_res_* = 12 h, and a randomly-selected 10% of the cells entering in the first 48 hours were tagged. Transit times were recorded for these cells on exiting the paracortex. The transit time distribution for the base case, with no cells subject to exit chemotaxis is shown in Fig. 7, where it is compared with the distribution generated using the influx rate corresponding to *T_res_* = 24 h. To explore the influence of attraction to the exit portals the tagged cells in the *T_res_* = 12 h case were made subject to chemotaxis, and in simulations with *K_E_* ranging from 1.0 to 5.0, the transit times of tagged cells were recorded. Residence times for these cases are plotted in Fig. 8A, and the distributions of transit time are shown in Fig. 8B. In these runs the probability of egress of a cell that is in the neighbourhood of an exit portal was fixed at 0.02, but it is possible that if a subset of cells experience chemotactic attraction to the exits, these cells should also have a greater chance of egress when close to a portal.

**Figure 9:**
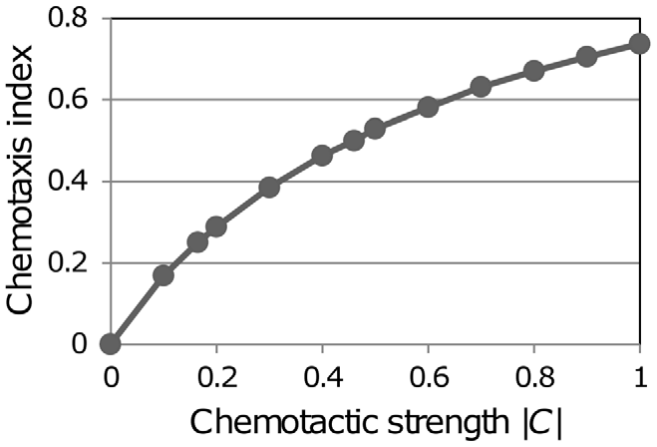
Dependence of chemotaxis index on the chemotactic strength |*C*|. Simulations were conducted to investigate the motion of a cell subject to a uniform (non-spatially varying) chemotactic influence ***C***. The chemotaxis index (CI) is the ratio of the straight-line displacement in the direction of ***C*** to the path length, for 10 steps on the lattice (this is a modification of the McCutcheon index). The chemotactic strength |***C***| was varied between 0 and 1, and in each case the CI was determined as the average of 1000 simulations.

For the purposes of comparison with experimentally observed chemotaxis, it is useful to have a measure of the chemotaxis generated numerically by this method. A commonly-adopted measure is the McCutcheon chemotaxis index, which is the ratio of a cell’s straight-line travel distance to path length, for a specified time interval [59], [60]. This measure is typically used in conjunction with an assay (e.g. on an agar plate or with a Zigmond chamber) in which cell motion is constrained to 2D in a layer within which the concentration gradient is fairly uniform. Quantification of T cell chemotaxis towards an attracting point in 3D presents several complications. First, the high level of “random walk” motility of the cells introduces a significant degree of variability in the results. Second, the strength of attraction varies strongly with distance from the attracting point, and a cell can spend long periods in regions where the randomness of its normal motion swamps any chemotactic effect. Third, crowding at the attracting point inevitably limits the ability of a cell to approach the point. Sidestepping the difficulties that these issues present, we have opted to estimate a chemotactic index (CI) for an artificial situation in which the chemotactic influence (conveyed by the chemotactic attraction vector ***C***) is spatially invariant. The direction of the vector ***C*** was arbitrarily set at (1,0,0), i.e. parallel to the X axis, and the magnitude was set to a value between 0 and 1 - varying the magnitude of ***C*** is equivalent to varying either *K_E_* or the distance from an exit. To estimate the CI for a cell with a given value of |***C***|, the motion of the cell on the lattice, combining random walk and chemotaxis, was simulated for a specified number of steps, and the average value was determined from a large number of simulated trajectories (1000). It was immediately apparent that the standard McCutcheon formula yields misleading results when the chemotactic effect is small compared with the cell’s normal persistent random walk motion. In the limit of no chemotaxis the apparent average CI is 0.53 for trajectories of 10 steps, 0.39 for 20 steps. To ensure that the CI goes to zero with |***C***|, the measure was changed to the distance travelled in the direction of attraction divided by the path length (in the limiting case of ***C*** = 0 there is no attraction, but selecting any fixed direction leads to the same result.) Another benefit of this change is that the results for 10 and 20 steps are virtually identical. With this small change the CI value ranges from 0 to 0.74 as |***C***| goes from 0 to 1. The results shown in Fig. 9 can be translated into the chemotactic effect at a distance *r* from an exit, for the bounded inverse square relation 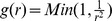, through 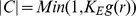. The maximum CI value of 0.74 applies when *r* ^2^< *K_E_*, e.g. if *K_E_* = 3, for *r* <1.73. The CI is 0.5 when *K_E_*/*r* ^2^ = 0.46, i.e. for *r* = 2.55 if *K_E_* = 3, and the CI is 0.25 when *K_E_*/*r* ^2^ = 0.165, i.e. for *r* = 4.26 if *K_E_* = 3. (Note that the distance *r* is the number of sites, where the site spacing is 6.3 µm.).

## Discussion

### Improved Treatment of T Cell Ingress and Egress

A method for simulating T cell trafficking has been presented, for an on-lattice model of T cell motility and trafficking within a lymph node paracortex, accounting for the dense packing of cells. It is assumed that cells leave the paracortex at discrete exit portals. The number of portals is determined by the cell population, the specified probability of egress from a neighbour site of an exit portal, and the specified residence time, according to an empirically-derived formula, and portal locations are fixed as long as the population does not change. This treatment of egress has been shown to maintain a steady-state cell population when the cell influx rate is constant. The approach was developed initially for the case of no chemotaxis, in which the arrival of a cell at an exit portal is determined solely by its random-walk motility.

To simulate the dramatic changes in lymphocyte ingress that occur during an immune response, driving first the expansion then the contraction of the T cell population, a sub-model has been developed to link influx rate to inflammation signals, by way of changes in vascularity modulated by growth factors. Reflecting the level of uncertainty about how lymph node blood vessels (in particular high endothelial venules) change in response to infection, no precise physiological meaning is attached to the term “vascularity” – it is simply treated as a factor that multiplies the baseline uninfected rate of influx. The inflammation-vascularity sub-model makes no pretense to faithfully representing biological mechanisms, but it does enable the crucially-important phenomenon of LN expansion-contraction to be taken into account in our model for the paracortical immune response. The model uses the assumption that the number of exit portals adjusts constantly in response to the changes in the cell population that occur during an immune response, in the no-chemotaxis case always satisfying Eq. 4, i.e. keeping the number of portals at the level corresponding to steady-state conditions with the current population. The portals move as the paracortical population varies, maintaining their locations near the boundary. Combining this treatment of cell egress with the sub-model linking inflammation to influx leads to a credible variation in the paracortex T cell population over the course of an immune response, in particular satisfying the important requirement that with the cessation of inflammation signals the population returns to the steady-state pre-infection level.

### A Novel Method for Simulating Chemotaxis

Chemotactic cues undoubtedly play a significant role in the behaviour of lymphocytes in the lymph node, and since the on-lattice approach has computational advantages when it is necessary to simulate large numbers of cells, a way of handling chemotaxis in this context was needed. We have proposed and demonstrated a method in which modifications to the cell’s “jump” probabilities depend on the strength and direction of the chemotaxis influence vector. In the case of a single chemokine, the influence vector is the chemokine concentration gradient, but a very useful feature of the method is that it allows the influence of multiple chemokines to be accounted for by simply taking the vector sum of the individual influence vectors. As demonstrated in the videos of simulated T cells under chemotactic influence, this method produces distinctive motility patterns, where T cells “drift” towards a chemokine source while maintaining their random walk motility. This drift is scalable, and is still exhibited by a sub-population of T cells that are responsive to chemotactic signals even when surrounded by a crowd of unresponsive T cells undertaking random walks. It has been pointed out that a slight directional bias in random-walk cell motion may be difficult or impossible to detect in the videos typically captured by intravital microscopy, but that such a bias level can still have a significant chemotactic effect over times much longer than the typical experiment’s duration [8].

We have used the action of chemotaxis in attracting cells towards exit portals as a convenient method for testing the chemotaxis sub-model. We used a simple function of cell’s position relative to the exit portal to compute the influence of a chemotactic agent sourced at each exit. Extensive simulations were employed to quantify the inverse relationship between the number of exit portals and the strength of chemotactic attraction, based on the requirement that the model maintains a steady-state cell population when cell influx is held constant. The simulations showed that chemotaxis has a marked effect when a subset of cells is susceptible – the residence time of these cells is much reduced.

However where chemotaxis acted on all cells in a crowded environment, the effects of that chemotaxis were subject to rapid saturation, as spatial constraints reduced the ability of new cells to access the exit portal. In our simulations this functional saturation occurred despite the continued stochastic motility of cells near exit portals; although there was constant flux through the sites adjacent to the exit portals, the probability of egress for all cells in those sites was fixed, so once all the sites were occupied, total egress rates were saturated. Under the circumstances we simulated, T cells effectively redistribute within the blob, from a uniform distribution to one where they are more crowded around exit portals, and correspondingly less crowded at other sites. If such circumstances prevailed in vivo – chemotactic influences affecting all cells equally, where all available space was not occupied by cells – then similar zones of crowding around the sources of chemotactic factors might be observed. In this context, with a large number of cells experiencing chemotaxis, it becomes an inescapable requirement that the method of simulation does not treat each cell’s motion as independent – spatial exclusion effects must be included in the model. Such spatial constraints would be automatically enforced in a comprehensive model of intercellular dynamics, but the computational demands of such a model may make it infeasible for simulation of cell populations of the order of 10^5^. Our model does not have the attractive simplicity and ease of implementation of an approach in which cell trajectories are simulated as if there were no cell-cell interactions [11], and neither does it enforce spatial exclusion as rigorously as a cell dynamics model [12], but it represents a practical solution to the problem of simulating the interaction of large numbers of cells in lymphoid tissue.

### Caveats

Models of complex biological phenomena are necessary simplifications and it is important to recognize the caveats involved in any model. In particular, models often need to make certain assumptions where definitive biological data are lacking. However so long as these assumptions are made overt in the model’s description, they can be updated as new data become available. Indeed the parameters that need to be estimated in a model often provide important pointers to measurements at which experimentation can fruitfully be directed.

Clearly the spherical blob in our simulations is not a scaled version of the whole paracortex. Structurally it lacks the fibroreticular network of the real paracortex, and it simplifies both entry and exit locations and their structure. While entry sites are becoming better defined through studies of the HEVs, the existence of exit portals has not been conclusively established, and the structure of the lymphatic sinuses they lead to is still understudied. At the functional level, the processes that cause T cell influx to vary so widely during an immune response need clarification, especially the precise nature of the vascular response, and how it is controlled.

Nevertheless, our model does efficiently capture many important aspects of reality, and although the limitations of such a “cartoon” representation must always be kept in mind, it does represent a working three-dimensional model that integrates both molecular and cellular data, and can readily be updated as new data come to hand. Data that would be particularly helpful in refining the model include: structural information regarding the 3D topography of functional ingress sites (HEVs) and egress sites into lymphatic sinuses; the nature of the vascular re-modeling that occurs during an immune response, and its molecular drivers; and measurement of the effects of chemotactic factors on the motility of T cells within LNs.

### Future Development

To avoid complicating the picture unduly, the processes of activation and proliferation that follow from T cell encounters with DCs bearing their cognate antigen have been omitted from the simulations reported here, but the methods presented have been incorporated into our agent-based model for the T cell immune response in the lymph node, in which T cell-DC interactions are simulated. The model makes possible investigations into many aspects of T cell activation and proliferation. Incorporation of chemotaxis into the simulation of lattice-based cell motility opens the way for computational exploration of the multiple influences of chemokines on lymphocytes in the lymph node. One obvious application will be to explore the possible role of chemotaxis toward DCs. We also plan to develop a sub-model that links T cell activation, the expression of CD69 and S1PR1 receptors, S1P chemotaxis and cell egress, in order to advance understanding of an important determining factor in the T cell immune response. Chemotaxis is known to have a major influence in the activation of B cells and formation of the germinal center, and we have initiated development of a model for processes in the B cell follicle. The ultimate objective is a combined model for B and T cell activation in the lymph node paracortex and follicle.

## Supporting Information

Video S1 **Motion of tagged cells in the case of no chemotaxis (** ***K_E_*** **= 0).** Cell motion was simulated in a spherical blob of 10 k cells with a single exit portal at the centre. 100 cells initially within a distance of 7 grids from the exit site were tagged and their motion tracked for four hours. (Note that the region is packed with cells that are not visualized.)
(MP4)

Video S2 **Motion of tagged cells subject to chemotaxis with** ***K_E_*** **= 1.** Cell motion was simulated in a spherical blob of 10 k cells with a single exit portal at the centre. 100 cells initially within a distance of 7 grids from the exit site were tagged and made subject to exit chemotaxis. The motion of the tagged cells was recorded over four hours. (Note that the region is packed with non-chemotactic cells that are not visualized.)
(MP4)

Video S3 **Motion of tagged cells subject to chemotaxis with** ***K_E_*** **= 2.** Cell motion was simulated in a spherical blob of 10 k cells with a single exit portal at the centre. 100 cells initially within a distance of 7 grids from the exit site were tagged and made subject to exit chemotaxis. The motion of the tagged cells was recorded over four hours. (Note that the region is packed with non-chemotactic cells that are not visualized.)
(MP4)

Video S4 **Motion of tagged cells subject to chemotaxis with** ***K_E_*** **= 3.** Cell motion was simulated in a spherical blob of 10 k cells with a single exit portal at the centre. 100 cells initially within a distance of 7 grids from the exit site were tagged and made subject to exit chemotaxis. The motion of the tagged cells was recorded over four hours. (Note that the region is packed with non-chemotactic cells that are not visualized.)
(MP4)

Video S5 **Motion of tagged cells subject to chemotaxis with** ***K_E_*** **= 4.** Cell motion was simulated in a spherical blob of 10 k cells with a single exit portal at the centre. 100 cells initially within a distance of 7 grids from the exit site were tagged and made subject to exit chemotaxis. The motion of the tagged cells was recorded over four hours. (Note that the region is packed with non-chemotactic cells that are not visualized.)
(MP4)
